# Atropine in Poisoning by Aconite

**Published:** 1890-06-14

**Authors:** 


### ATROPINE IN POISONING BY ACONITE.

We were recently called in to a case of accidental poisoning by aconite, a lad of twelve, naturally weak, with feeble action of the heart, and convalescing from illness caused by sewer gas, having taken ʒii. of the tincture in mistake for a sleeping draught. The family attendant had given him 40 grains of sulphate of zinc, which caused violent vomiting before he sent for us. The boy was pale and prostrate, his lips blue, and his body bathed in perspiration. The pulse was 56, and so compressible as to disappear under the least pressure. We at once gave a hypodermic injection of $\frac{1}{100}$ grain of atropine. The natural colour returned to the face and lips almost immediately; the pulse gained in strength and frequency, until within half-an-hour it had reached 136, and was full and bounding. The pupils were, of course, dilated, the throat dry, the skin natural, and the feeling of faintness had passed away. The excessive heart's action then slowly subsided, until, when we left him at the end of three hours, it was beating at the rate of 80 to the minute, and with normal, or for him somewhat unusual strength. He slept comfortably, and was next day none the worse for his adventure.

